# Dynamics of Glycoprotein Charge in the Evolutionary History of Human Influenza

**DOI:** 10.1371/journal.pone.0015674

**Published:** 2010-12-30

**Authors:** Nimalan Arinaminpathy, Bryan Grenfell

**Affiliations:** 1 Department of Ecology and Evolutionary Biology, Princeton University, Princeton, New Jersey, United States of America; 2 Fogarty International Center, National Institutes of Health, Bethesda, Maryland, United States of America; Institut Pasteur, France

## Abstract

**Background:**

Influenza viruses show a significant capacity to evade host immunity; this is manifest both as large occasional jumps in the antigenic phenotype of viral surface molecules and in gradual antigenic changes leading to annual influenza epidemics in humans. Recent mouse studies show that avidity for host cells can play an important role in polyclonal antibody escape, and further that electrostatic charge of the hemagglutinin glycoprotein can contribute to such avidity.

**Methodology/Principal Findings:**

We test the role of glycoprotein charge on sequence data from the three major subtypes of influenza A in humans, using a simple method of calculating net glycoprotein charge. Of all subtypes, H3N2 in humans shows a striking pattern of increasing positive charge since its introduction in 1968. Notably, this trend applies to both hemagglutinin and neuraminidase glycoproteins. In the late 1980s hemagglutinin charge reached a plateau, while neuraminidase charge started to decline. We identify key groups of amino acid sites involved in this charge trend.

**Conclusions/Significance:**

To our knowledge these are the first indications that, for human H3N2, net glycoprotein charge covaries strongly with antigenic drift on a global scale. Further work is needed to elucidate how such charge interacts with other immune escape mechanisms, such as glycosylation, and we discuss important questions arising for future study.

## Introduction

The influenza A virus is a prominent example of a pathogen capable of adapting to evade host immunity [1], [2], with profound implications for epidemiology and control. Hence, for example, seasonal influenza vaccines need to be updated frequently, to maintain protection in risk-groups against currently circulating strains [3].

Immunity to influenza is mediated predominantly by neutralizing antibodies raised against two surface glycoproteins, hemagglutinin (HA) and neuraminidase (NA) [4]. Antigenic ‘drift’ refers to the incremental adaptations in these antigens from year to year, forming the basis for immune evasion. However, while phenomenological characteristics of drift have been well-characterised [5], [6], the key molecular and evolutionary determinants of immune escape remain incompletely understood.

Amino acid substitutions directly reducing or abolishing antibody recognition of major epitopes are likely to be the predominant part of immune escape [7]. Glycosylation - the post-transcriptional acquisition of carbohydrate side-chains - can have the additional effect of ‘masking’ epitopes from antibody recognition [8], but at a cost: heavily glycosylated viruses are also more susceptible to capture by the host innate immune system [9].

A recent study [10], using challenges of vaccinated and naïve mice, has highlighted another, potentially significant element of the immune escape repertoire: the selection of ‘adsorptive mutants’, showing enhanced binding to target cells. Demonstrating a strong association between immune escape and avidity for target cells, this study suggests that viral replication success depends on a competition between host-cell binding, and engagement by neutralizing antibodies. Conversely, however, naïve mice show positive selection for reduced avidity, indicating that this strategy of immune escape also carries a fitness cost – this may be due, for example, to high-avidity progeny virions being released less efficiently from host cells.

A notable aspect of avidity demonstrated in this study is that of electrostatic charge – several immune escape mutants show an increased positive charge in HA. From a molecular standpoint, since host cell receptors possess a strong negative charge, virions with a higher net positive charge may tend to promote favourable electrostatic interactions with target cells [11].

The role of electrostatic charge has been appreciated for other human viruses: a notable example is HIV, where a viral phenotype associated with rapid progression to AIDS is distinguished from those associated with early stages of HIV-infection by the presence of charged residues at two amino acid positions on the V3 region of the gp120 subunit [12]. However, the potential relevance of charge to influenza viral dynamics has so far received comparatively little attention.

Thus motivated, we ask the following questions: *considering epidemic influenza in humans, is there any indication for the role of electrostatic charge in viral evolution? How might these relate to observed patterns of antigenic drift, most notably for the subtype H3N2? Specifically (in line with* [*10*] *, is increasing immune escape during antigenic drift associated with an increase in viral surface charge?* We address these questions by exploring secular changes in the net charge on HA and NA molecules revealed by amino acid sequences from published influenza databases. We present a striking pattern of coherent charge accumulation. In contrast with the HIV-V2 protein this pattern involves at least 20 amino acid sites across the length of the hemagglutinin sequence, with a correspondingly greater degree of net charge change accumulated over several decades. Overall, while these observations are not definitive of the role of electric charge in immune escape, they cast light on intriguing, hitherto unreported phenomena, calling for further experimental study. We discuss important questions arising for further investigation.

## Methods

At physiological pH, protein-associated amino acids are generally charge-neutral, with five exceptions: Arginine, Histidine and Lysine are ionized with a single positive net charge, while Aspartic acid and Glutamic acid are ionized with a single negative net charge [13]. Given the amino acid sequence for a certain glycoprotein, we may then calculate overall net charge, to a first approximation, simply by summing charge contributions from all of these constituents. In the discussion section we return to qualify the assumption of physiological pH for all amino acids in the sequence.

Sequences for HA and NA were obtained from the NIAID influenza database [14], with the restriction of being full-length to avoid potential biases due to variable sampling of amino acid positions. Identical sequences in the same year were collapsed to a single one, to avoid biases due to over-representation of certain samples in the database. Net glycoprotein charge was then calculated for each sequence, using the approach described above.

## Results

**Figure 1:**
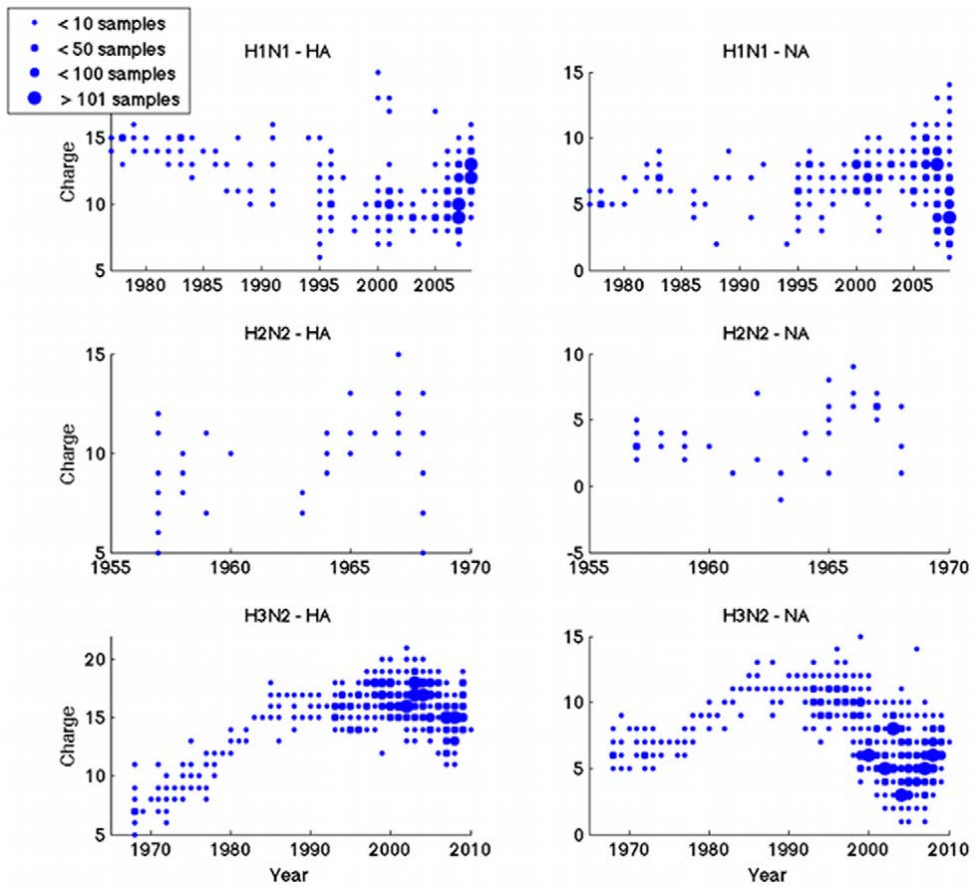
Timecourses of total charge of the major antigens HA and NA. Shown for three main human subtypes of influenza A. Sizes of dots represent frequencies, as indicated in the legend. Increase in positive charge for both H3N2 glycoproteins is statistically significant (p<0.0001 in both cases).

Figure 1 shows charge plotted against year of sampling, for the three major subtypes of human influenza: H1N1 (1977–2008), H2N2 and H3N2. There is no apparent pattern for the first two. For the H3N2 subtype, however, charges for both HA and NA show clear increasing trends following introduction of this virus in the Hong Kong pandemic of 1968. Intriguingly this trend changes for both HA and NA in the same year, in 1986. After this point HA charge appears to plateau, while NA charge undergoes a noisy decline.

A potential difficulty with this data set is the variability of viral sampling through time, as illustrated by the size of the points in figure 1 and as plotted in Figure S1. In particular, there is a paucity of full-length sequences collected in the mid-1970s. Nonetheless, a linear regression of charge against year offers a simple approach for testing the statistical support for an increasing HA charge, while allowing for widely varying sample sizes from year to year. Such an approach yields a significance level of p<0.0001, for both HA and for NA. An alternative approach is to choose just two years for comparison, using a t-test to assess support for a statistically significant difference in mean charge from the first timepoint to the second. Applying this to data from 1968 (23 samples) and 1993 (32 samples) once again gives a significance level of p<0.0001, for both HA and NA. Despite the patchiness of data, therefore, there is sufficient evidence to suggest a significant increase in charge between 1968 and 1992.

**Figure 2:**
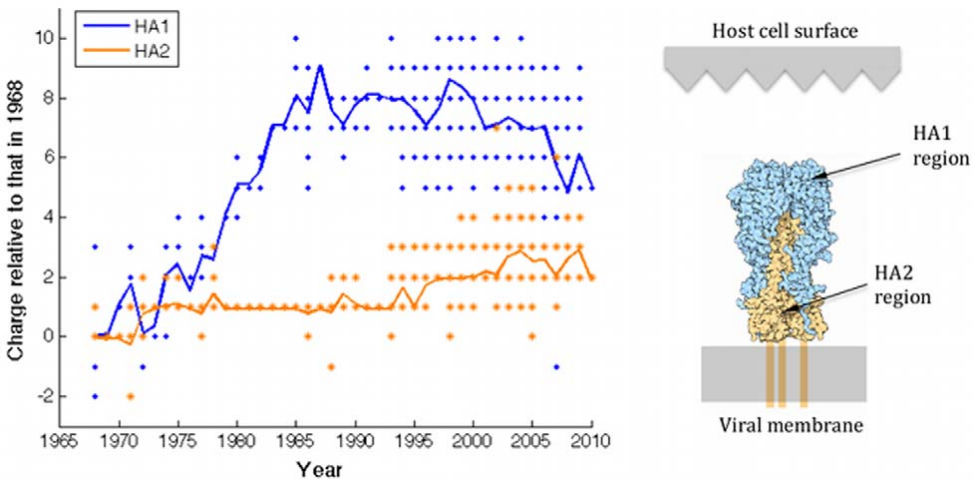
Comparison of changes in charge in the two subunits of the H3N2 HA molecule. Solid lines indicate mean trends. The side-bar illustrates these regions on the HA molecule (diagram source: RCSB Protein Data Bank).

The HA monomer is an assembly of two subunits: HA1 contains the globular head of the HA molecule, while HA2 contains the stalk region, and is attached to the viral membrane. To explore whether changes in charge occur preferentially in either of these subunits, the procedure above is repeated for the relevant segments of the HA amino acid sequence. Results shown in Figure 2 indicate that the bulk of the charge changes occurred in the HA1 subunit, as might be expected: containing the receptor binding site, this region is most directly involved in attachment to host cells. Thus (as predicted by [10]), H3N2 charge increases in parallel with net antigenic escape, at least over the early part of the virus's history in humans.

**Figure 3:**
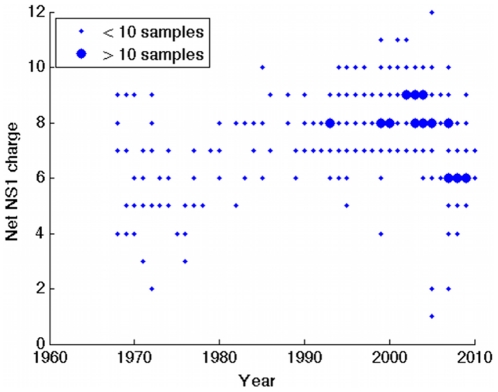
Charge data for the influenza NS1 protein. Restricted to the H3N2 subtype.

It is, of course, possible that this observed trend is only a by-product of HA evolution, without any independent antigenic relevance. To explore this possibility the same charge analysis was applied to NS1 [15], another influenza viral component showing rapid evolution [16], but without the antigenicity of HA or NA in natural infection. NS1 is expressed as a protein only in the intracellular stage of the replication cycle, with the function of antagonising host innate antiviral responses, as well as playing an important role in regulating transcription. Figure 3 plots charge variation of NS1 restricted to human H3N2, showing no significant temporal trend, despite the rapid evolution of this viral component. Hence it would appear that the coherent temporal trends observed for HA and NA are more than simply a by-product of protein evolution.

Returning to the HA1 subunit of Hemagglutinin, we ask which set of amino acid positions accounts for the most temporal variation in charge, with a view to comparing these against known features of H3N2 evolutionary history. As noted earlier (see Figure S1), the number of amino acid sequences available from GenBank is itself variable in time. To avoid artefacts from years with little or no viral sampling we restrict attention to a comparison between samples in 1968, 1985 and 2000, timepoints having 45, 35 and 249 samples respectively.

Figure S2 plots the estimated probability of an amino acid site showing positive or negative charge, plotted along the length of the HA1 sequence in 2000. With three exceptions, sites tend to show clearly dominant charge states (whether positive, neutral or negative) and charge transitions at given sites tend to be characterized by population-level ‘switches’ from one state to another. This is perhaps not surprising, given the comparatively limited population-level diversity of influenza in any given year [17].

As one possible partitioning of amino acid sites, those sites that have undergone a change in charge state from 1968 to 2000 are designated as ‘group 1’. Further, we define ‘group 2’ sites as those showing a change in charge between 1968 and 1985, followed by a reversion between 1985 and 2000, thus resulting in zero net charge difference over the whole time period. Amino acid sites belonging to these two groups are summarized in table 1.

**Table 1:** Amino acids of the HA1 sequence involved in changes of charge state when comparing 1968, 1985 and 2000.

20001968	Positive (+)	Neutral	Negative (−)
Positive (+)	—	57/156	—
Neutral	142/145/155/158/160/173/276	*189/197*	53/62/83/188
Negative (−)	2/82	31/63/275	*172*

‘Group 2’ amino acids, as defined in the main text, are shown in italics on the diagonal – all three exhibited an increase in charge of +1 in 1985, followed by a reversion before 2000. All other amino acids belong to ‘group 1’. Note that those elements below the diagonal contribute to a positive overall charge, and vice versa for those above the diagonal.

**Figure 4:**
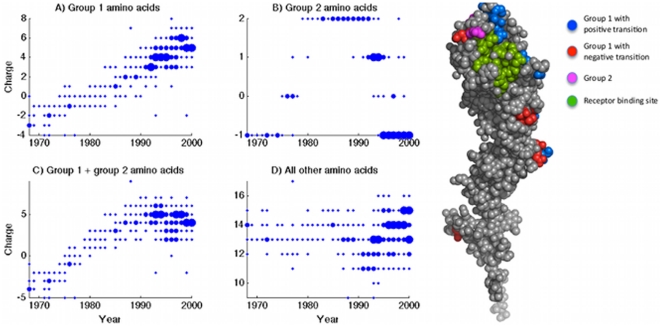
Charge trends when summed separately over ‘group 1’ and ‘group 2’ amino acids (see **table 1**). This approach shows that certain sites, which reverted charge state between 1985 and 2000, may account for the ‘plateau’ in total HA charge observed over this time. The right-hand panel locates the amino acids on the HA structure, showing the receptor binding site in green; group 1 positions showing a positive transition (in blue); group 1 positions showing a negative transition (in red); and group 2 positions, showing a transition followed by a reversion in a subsequent year (in magenta).

Figure 4 plots charge trends when summed separately over these sets of amino acids, showing clear qualitative and quantitative differences between them. Group 1 amino acids together show a continuous, steady increase in total charge, accounting for the greatest temporal variation between 1968 and 2000. However, panel C demonstrates that incorporating group 2 amino acids accounts for the plateau in total HA charge reached in the early 1990s. The lack of temporal variability in panel D suggests that groups 1 and 2 together capture the essential secular charge variation shown by the whole HA molecule. While it remains unclear what biological relevance – if any – these groupings may have, this basic analysis points to specific amino acid positions that could be relevant in explaining the change in charge trend in the late 1980s.

The right-hand panel of figure 4 shows a visualization of group 1 and 2 amino acid positions on the HA1 structure [18]. A notable feature is the clustering of several of these positions around the receptor binding site (shown in green), the active site for binding with target cell sialic acid. As we might expect, most of these are group 1 positions undergoing a positive charge transition (shown in blue) between 1968 and 2000. Conversely, sites undergoing a negative transition (shown in red) are – with one exception – all further down the HA length.

### Comparison with known mechanisms of immune escape

A natural hypothesis arising from [10] is that H3N2 charge covaries with other immune escape mechanisms already known to have been significant in H3N2 evolutionary history. We now explore possible connections with two notable features of H3N2 evolution, namely punctuated antigenic drift, and the accumulation of glycosylation sites.

H3N2 antigenic drift is characterized by stepwise antigenic changes that occur every 2–8 years [5], against a background of a relatively steady rate of amino acid substitutions. Thus H3N2 viruses show grouping into antigenic ‘clusters’, contiguous but overlapping in time. Amino acid substitutions in HA associated with transitions from one cluster to another have previously been identified: calculating the charge contribution from each of these over time (table 2) shows that charge effects ranged from −2 to +3. However, the net change arising from antigenic cluster transitions since 1968 is +1, accounting for only a small part of the total increase of almost +10 that occurred throughout HA during this same period. Thus, on an aggregate level, there is no simple connection between charge and cluster transitions.

**Table 2:** Amino acid substitutions accompanying antigenic jumps, and their associated charge changes (see [5]).

Year	Amino acid substitutions	Overall charge effect
1972	T122N; (*G144D*); T155Y; (*N188D*); R207K	2 −
1975	N137S; S145N; L164Q; **Q189K**; (*S193D*); (*N53D*); I278S; F174S; R102K; I213V; I217V; I230V	1 −
1977	S137Y; (*G158E*); Q164L; **D193N**; K50R; **D53N**; S174F; K201R; V213I; V230I; **E82K**; M260I	3 +
1979	N133S; P143S; G146S; (*K156E*); **T160K**; **Q197R**; (*N53D*); N54S; **D172G**; V217I; V244L; **162K**; (*K82E*)	1 −
1987	(*G124D*); **Y155H**; K189R	0
1989	**N145K**	1 +
1992	(*S133D*); (*K145N*); **E156K**; E190D	0
1995	**N145K**	1 +
1997	(*K156Q*); E158K; V196A; **N276K**; (*K62E*);	0
2002	A131T; (*H155T*); **Q156H**; (*R50G*); (*H75Q*); **E83K**; L25I; V202I; **W222R**; (*G225D*)	0

While successive antigenic groups overlap in time, ‘year’ denotes the timing of the first vaccine strain incorporating the given substitutions. Substitutions shown in bold denote a net positive change, and vice versa for those in parentheses.

On the level of individual amino acid positions, there are nonetheless some suggestive results: all group 2 amino acids are also implicated in cluster transitions, with their charge switches coinciding with the timing of those jumps. About half of group 1 amino acid sites are involved in cluster transitions. Of these, site 145 is prominent for having the potential to cause unusually large antigenic change, being the sole site involved in two separate cluster transitions [5], in both cases undergoing an increase in positive charge (see table 2). However, further work is needed to elucidate the significance – if any – of these apparent connections.

Another unique feature of human H3N2 evolutionary history is the steady accumulation of potential N-linked glycosylation sites through time. Such positions are characterised by NXS/T sequons, with X being any amino acid other than Proline. Human H3N2 is unique among influenza A subtypes for accumulating potential glycosylation sites since 1968 [19], [20], with evidence that this is in response to selection pressure [21]. There are indeed tangible reasons to expect that glycosylation may interact with charge locally on the HA surface: glycans positioned near the receptor binding site can lower affinity for host cells [22]–[24] and thus increasing positive charge near the glycosylation site could have a compensatory role by mitigating this effect. Alternatively complex glycans may themselves carry a negative charge, by virtue of attached sialic acid, whose effect could again be mitigated by the presence of positive charges near the glycosylation site.

**Figure 5:**
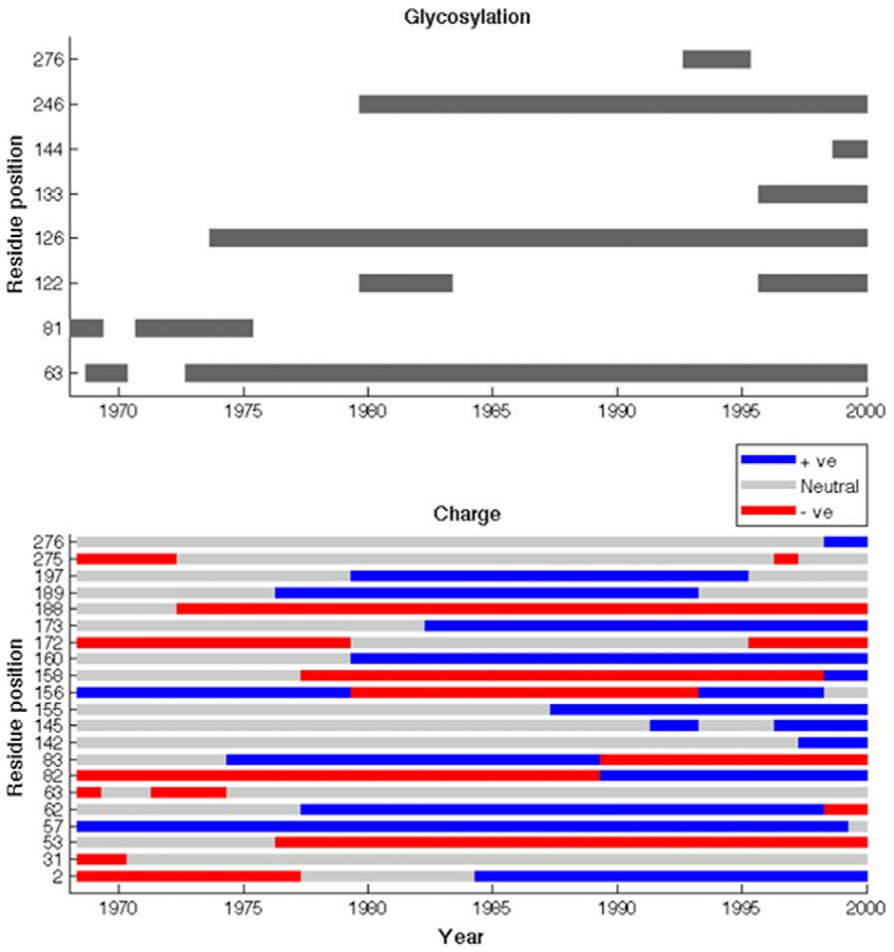
Estimated timing of major charge and glycosylation events. In the lower panel, grey bars denote the presence of an N-glycosylation sequon in at least 10% of sequences in a year. Note that where there are only few samples in a year, as depicted in Figure S1, estimates in both panels become less reliable.

Figure 5 shows the approximate timing of acquisition and loss of glycosylation sequons in HA1 sequences, comparing against the dominant charge states of group 1 and 2 amino acids. In accordance with [19]–[21], the upper panel shows a steady increase in the number of glycosylation sites with time. There are indeed amino acid sites that underwent both a positive charge transition and the acquisition of a potential glycosylation site, such as 63, 276, and the region 81–83. However, such events at these sites are not necessarily synchronous, most apparently being separated by several years, and thus there is no clear causal connection. Further data is needed to explore the possibility of more subtle connections between glycosylation and charge.

We can extend this general analysis to glycoprotein charge in non-human hosts, as detailed in the Appendix. In particular, Figure S3 suggests that H3 HA charge in avian hosts has been relatively stable since 1970, at a level in agreement with that of human H3 in its introduction in 1968. This is indeed as we might expect, given the avian origin of H3 in humans. Figure S4 broadens this search to other subtypes and host species, illustrating that none show a coherent pattern comparable to human H3N2.

## Discussion

Conformational changes in epitopic regions undoubtedly play an important – perhaps dominant – role in inhibiting binding by neutralising antibodies. Recent experiments show that the enhancement of avidity for host cells is also a potentially potent mechanism [10]. Here we have demonstrated clear indications that, in the specific case of H3N2, the virus has undergone increasing positive charge since its introduction in humans. Equally intriguing is the apparent uniqueness of this pattern, to H3N2 in humans. Indeed, it is perhaps no coincidence that human H3N2 is a paradigm of antigenic drift, showing an unrivalled tempo and extent of antigenic variation in comparison with other known influenza subtype/host combinations [4].

Some important caveats are in order here: first, there are alternative interpretations for the patterns we observe. For instance, it may well be the case that charge increments are only an incidental effect of substitutions whose primary antigenic impact is a conformational change at active sites. Related to this point, it must be noted that charge is not a necessary feature for increasing avidity, and is therefore only one of several potential immune escape strategies. With these provisions in mind, however, the key message of our results is simple: given the complexity and sheer multiplicity of interacting host and viral factors in viral immune escape, it is striking that such a simple measure of charge should yield such a marked pattern in the global database over several decades. Particularly in the light of previous results of the role of avidity in immune escape [10], we believe the phenomena we have demonstrated here form a basis for testable hypotheses warranting further investigation.

Regarding our simple algorithm for calculating net charge, an assumption requiring some care is that of amino acids being subject to physiological pH. While this is likely to apply for surface residues, those residues internal to the HA structure may instead be associated with significantly altered pKa values. Nonetheless, the HA1 structure shown in figure 4 indicates that such effects would not be expected to significantly alter our results. It illustrates that all group 1 and 2 amino acids are surface residues, and indeed summing charge over these positions alone reproduces the essential secular trend in figure 1 for the whole HA molecule.

The biological significance of these identified sites remains unclear. Moreover, at this point we can only speculate about connections between charge, glycosylation and antigenic clustering. It is relevant to note that there is yet no clear indication of a systematic relationship between H3N2 glycosylation and antigenic clustering [25]. Similarly, the simple comparisons we have conducted here do not yield any immediate relationships with charge. Clearly, however, this preliminary analysis addresses only the simplest hypotheses and says little about the possibility of more subtle interactions. For example, such interactions may be expressed on an aggregate scale: given the comparative sizes of virion and host cell it is possible that for most relevant dynamics the virion acts as a point charge on the physical scale of the cell, characterized more by its net charge than by the distribution of that charge on its surface. More detailed studies of virus-cell interactions in the presence of antibodies would clearly help to resolve questions like these. Furthermore, assays of binding kinetics of viral samples since 1968 will be essential in confirming whether the charge trend demonstrated here does in fact accompany an increase in H3N2 avidity for host cells.

A prominent, unexplained feature of H3N2 glycoprotein charge behaviour is the change in trend, for HA and NA alike, occurring in 1986. It is unclear whether this arises from a change in selection pressure, or from functional constraints on the virus – it is possible, for example, that an upper limit on the total virion charge is required for structural stability. Our preliminary analysis does not suggest any general, direct connection with observed antigenic cluster transitions for H3N2. It is, however, perhaps relevant that a cluster transition occurred in 1987, at about the same time as the shifts in the charge trends. This particular event is notable for at least two reasons: first, it marked the end of an unusually long period free of cluster transitions [5]. Second, in ‘antigenic space’ (as measured by hemagglutination inhibition assays with ferret sera), early antigenic variation proceeded in a predominantly linear fashion from the ancestral, 1968 strain. The transition in 1987 was unusual in breaking this linear trend for the first time [5], possibly showing higher cross-reactivity with previous strains as a result. If indeed relevant, both of these scenarios would point to a role of shifting selection pressure, in the changing trends in charge following 1987.

In addition to the issues raised above, several other potential avenues for future work arise from our observations. While the link between avidity and immune escape has been demonstrated [10], an important question for future experimental study is how these effects translate to viral *transmission* and hence fitness. A natural extension of existing work would be a study of serial infection or transmission, to allow the accumulation of immune escape mutations over many generations. Virological questions include: is there an inherent limit on the charge that a viral structure can support? How does a virus offset replication costs owing to greater avidity? Interestingly, there were no signs of co-adaptation of NA in [10]. Are there limits on NA function that would explain this?

In its entirety the influenza immune escape repertoire undoubtedly involves several mechanisms, and it is likely that these mechanisms interact. Ultimately, therefore, it is probable that the function of electrostatic charge, and avidity in general, can only be adequately understood in conjunction with other, parallel processes, such as glycosylation. Nonetheless, the prominent patterns we have discussed here point to a potentially significant role for glycoprotein charge, deserving further elucidation.

## Supporting Information

Figure S1 **Number of HA1 amino acid sequences available from GenBank, as a function of time.**
(TIF)

Figure S2 **The probability distribution of charge states occupied by each amino acid position in the HA1 sequence, in the year 2000.** The majority of points lie along the edges, indicating a strong dominance of the corresponding charge state at those sites.
(TIF)

Figure S3 **HA charge distribution in avian hosts, for H3 viruses.** The figure above was obtained using all 364 full-length avian H3 virus sequences in the NIAID influenza database [14]. Of these, the majority (349) lie in the charge range +4 to +9. Chickens, as poultry, are considered one of the possible routes of introduction of novel human influenza viruses. All sequences from chickens had charge from +6 to +7. There is a clear species-related structure in the remaining 15 samples: 11 were from turkeys, 1 from a northern pintail, and 3 from mallards. Conversely, sequences isolated from turkeys all had charges +10 or greater. An explanation for the consistent anomaly of turkey viruses is that most of these samples arose from two separate studies implicating swine in the introduction of H3N2 into turkey farms [26], [27]. As shown in Figure S4, the charge levels of HA in swine H3N2 in 2004 are in line with those observed in turkeys at about the same time, above.
(TIF)

Figure S4 **HA and NA charge distribution for two illustrative subtype/host species pairs.** Other subtypes, and hosts, show a comparable behaviour, with no discernible pattern over time.
(TIF)
