# Supplementary figures and images for: Dynamics of Glycoprotein Charge in the Evolutionary History of Human Influenza
Source: PLoS One. 2010 Dec 30;5(12):e15674. doi: 10.1371/journal.pone.0015674 (PMC3012697; doi:10.1371/journal.pone.0015674)

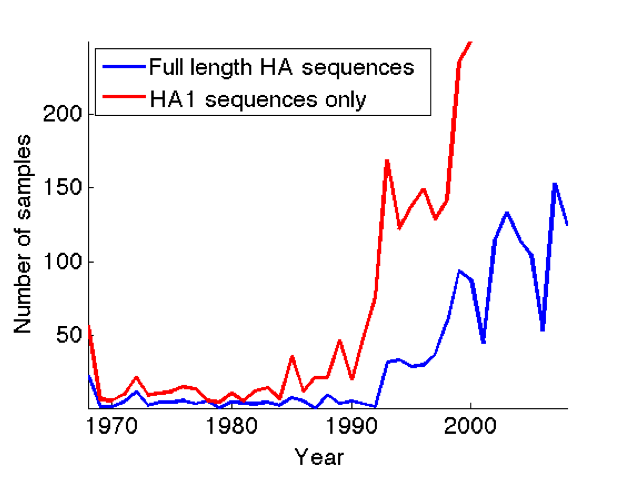

Supplement: Figure S1 — Number of HA1 amino acid sequences available from GenBank, as a function of time. (TIF) [file pone.0015674.s001.tif]

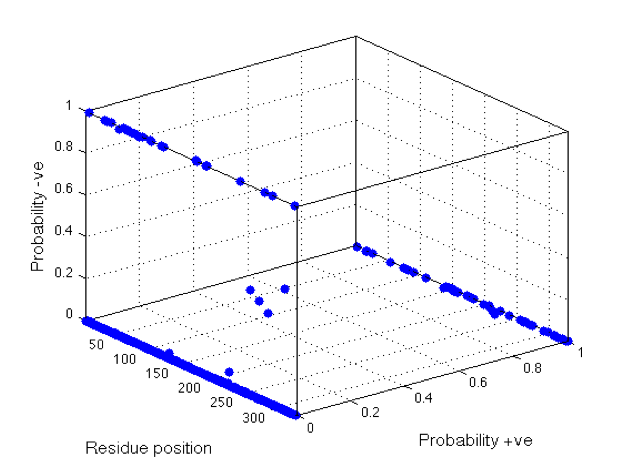

Supplement: Figure S2 — The probability distribution of charge states occupied by each amino acid position in the HA1 sequence, in the year 2000. The majority of points lie along the edges, indicating a strong dominance of the corresponding charge state at those sites. (TIF) [file pone.0015674.s002.tif]

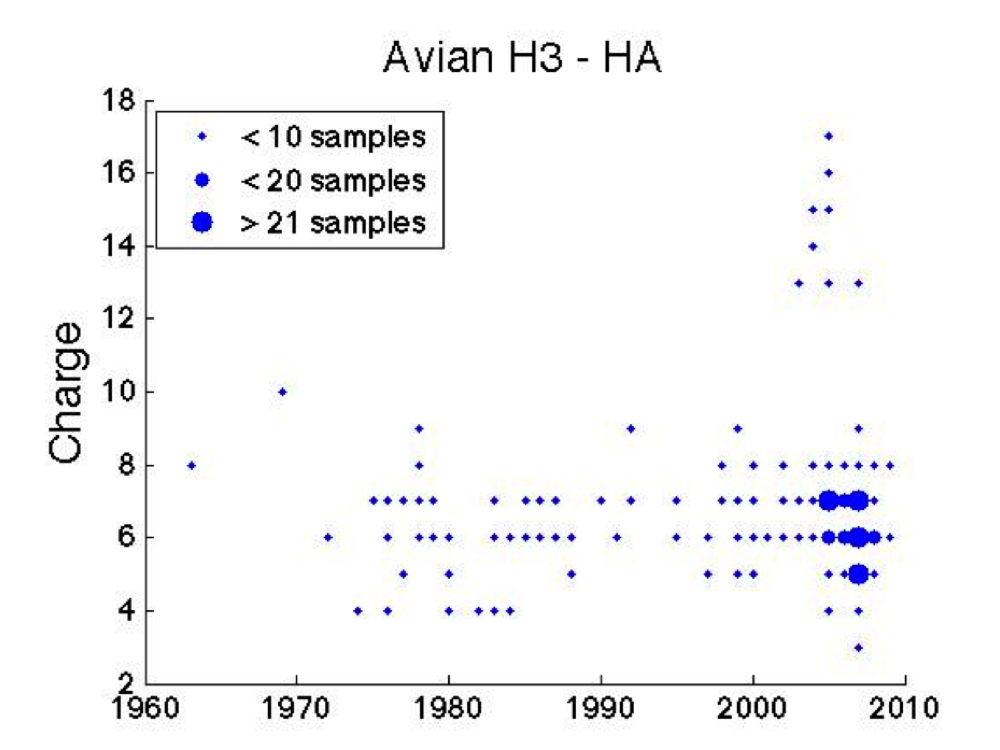

Supplement: Figure S3 — HA charge distribution in avian hosts, for H3 viruses. The figure above was obtained using all 364 full-length avian H3 virus sequences in the NIAID influenza database [14]. Of these, the majority (349) lie in the charge range +4 to +9. Chickens, as poultry, are considered one of the possible routes of introduction of novel human influenza viruses. All sequences from chickens had charge from +6 to +7. There is a clear species-related structure in the remaining 15 samples: 11 were from turkeys, 1 from a northern pintail, and 3 from mallards. Conversely, sequences isolated from turkeys all had charges +10 or greater. An explanation for the consistent anomaly of turkey viruses is that most of these samples arose from two separate studies implicating swine in the introduction of H3N2 into turkey farms [26], [27]. As shown in Figure S4, the charge levels of HA in swine H3N2 in 2004 are in line with those observed in turkeys at about the same time, above. (TIF) [file pone.0015674.s003.tif]

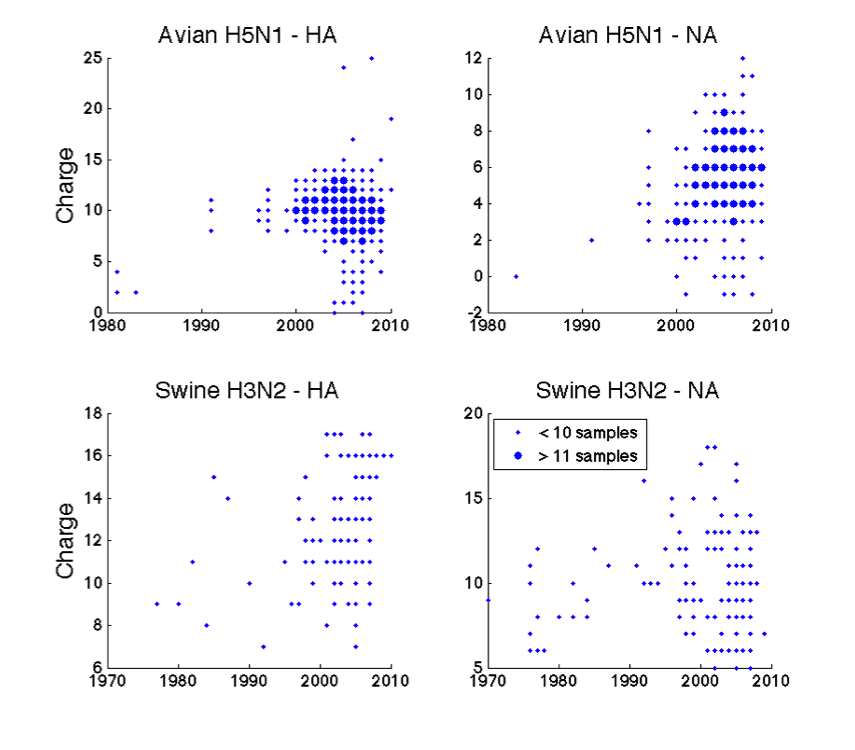

Supplement: Figure S4 — HA and NA charge distribution for two illustrative subtype/host species pairs. Other subtypes, and hosts, show a comparable behaviour, with no discernible pattern over time. (TIF) [file pone.0015674.s004.tif]
